# Anti-Tumor Effects of Mfn2 in Gastric Cancer

**DOI:** 10.3390/ijms140713005

**Published:** 2013-06-24

**Authors:** Ge-Er Zhang, Hai-Long Jin, Xian-Ke Lin, Chao Chen, Xiao-Sun Liu, Qing Zhang, Ji-Ren Yu

**Affiliations:** Department of Gastrointestinal Surgery, the First Affiliated Hospital, Medical College, Zhejiang University, Hangzhou 310003, China; E-Mails: zhanggeer@hotmail.com (G.-E.Z.); jhl1988@zju.edu.cn (H.-L.J.); wslxk0122@hotmail.com (X.-K.L.); chris19881228@hotmail.com (C.C.); xiaosunliu@hotmail.com (X.-S.L.); zhangqingzyyy@hotmail.com (Q.Z.)

**Keywords:** gastric cancer, Mfn2, biomarker, anti-tumor gene

## Abstract

Mitofusin-2 (Mfn2) is a mitochondrial outer membrane protein involved in mitochondrial fusion. Its mutation can cause Charcot-Marie-Tooth disease. Recent studies of Mfn2 in cancer research have not included gastric cancer. We confirmed that Mfn2 expression was lower in tumor tissue than in normal gastric mucosal tissue and that it was negatively correlated with tumor size, indicating an anti-tumor role for Mfn2. *In vitro* experiments showed that Mfn2 overexpression suppressed gastric cancer cell proliferation and colony formation, weakened the invasion and migratory ability of cancer cells by downregulating MMP-2 and MMP-9, halted the cell cycle and induced apoptosis. Western blotting indicated the likely involvement of P21 and PI3K/Akt signaling. Therefore, Mfn2 is a potential anti-tumor gene and a potential therapeutic target for treating gastric cancer. The progress of gastric cancer may be delayed by controlling Mfn2 expression.

## 1. Introduction

Gastric cancer is one of the leading causes of cancer-related mortality worldwide. It has been estimated that a total of 989,600 new gastric cancer cases and 738,000 deaths occurred in 2008 [1]. Countries in the Western Pacific Region have the highest reported incidence of gastric cancer; in a recent survey, 47% of new gastric cancer cases across the world occurred in China, and more than 90% of those cases were found to be advanced [2]. Surgery alone cannot guarantee good long-term survival for patients. Therefore, it is critical that we develop new therapeutic strategies, including molecule-targeted therapy.

Mitofusin-2 (Mfn2), a potential target molecule, has been extensively studied in Charcot-Marie-Tooth (CMT) disease (a heterogeneous group of inherited peripheral neuropathies). Point mutations in Mfn2 have been found in several types of CMT disease and are likely to be the fourth most common cause of CMT [3]. Mfn2 is a mitochondrial outer membrane protein that participates primarily in mitochondrial fusion [4]. It can also tether the ER to mitochondria and is required for efficient mitochondrial Ca^2+^ uptake [5]. Previously, it was known as hyperplasia suppressor gene (HSG). Studies of vascular proliferative disorders have demonstrated that the overexpression of HSG markedly suppressed serum-evoked vascular smooth muscle cell (VSMC) proliferation in culture and blocked balloon injury-induced neointimal VSMC proliferation and restenosis in rat carotid arteries [6]. Recently, Mfn2 has attracted interest in tumor research. Several studies have investigated the function of Mfn2 in different types of malignancies, including lung, liver and urinary bladder cancers; Mfn2 is thought to promote pro-apoptotic and anti-proliferative functions [7–9].

However, the mechanism underlying the anti-tumor effect of Mfn2 is unclear, and Mfn2 has never been studied in gastric cancer. In the current study, we examined the expression of Mfn2 in tissues from 90 gastric cancer cases and evaluated its prognostic significance in gastric cancer. Furthermore, we explored the cellular function and mechanism of Mfn2 *in vitro* using gastric cancer cell lines.

## 2. Results and Discussion

### 2.1. Results

#### 2.1.1. Higher Mfn2 Expression in Normal Mucosal Tissue than in Tumor Tissue

**Figure 1:**
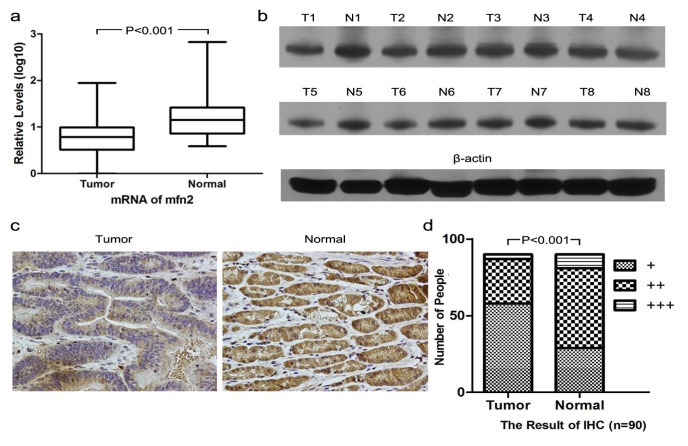
Mfn2 expression was confirmed higher in normal mucosal tissue than in tumor tissue by three methods, including (**a**) QRT-PCR (*n* = 30), (**b**) Western blot (*n* = 20, only showing eight pairs) and (**d**) IHC (*n* = 90). Representative IHC staining of Mfn2 in tumor tissue and normal mucosa tissue is shown in (**c**) (200×).

We analyzed the expression of Mfn2 in normal mucosal tissue and tumor tissue in gastric cancer patients using three different methods and obtained consistent results. We analyzed 30 pairs of samples by QRT-PCR (Figure 1a), 20 pairs by Western blotting (Figure 1b) and all 90 pairs by immunohistochemistry (Figure 1c,d). The expression was higher in normal tissue compared to that in tumor tissue. The difference was significant by both QRT-PCR and immunohistochemistry (*p* < 0.001). By Western blot analysis, the expression of Mfn2 in normal mucosal tissue was higher than that in tumor tissue for 15 patients, whereas the other five patients showed no obvious difference.

#### 2.1.2. Efficiency of Transfection

**Figure 2:**
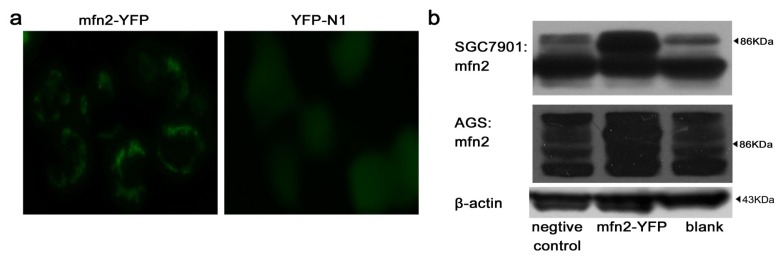
Transfection: (**a**) Gastric cancer cell line SGC7901 was transfected with Mfn2-YFP (**left**) or YFP-N1 (**right**); (**b**) Western blot analysis confirmed that the expression of Mfn2 increased significantly both in SGC7901 and AGS after they were transfected with Mfn2-YFP.

We studied the Mfn2 expression level in different gastric cell lines (AGS, KATO iii, MGC803, SGC7901, MKN28 and MKN45) by Western blotting at first. We selected AGS and SGC7901 as our main object of study, due to their low expression of Mfn2. Because Mfn2 is a mitochondrial outer membrane protein, we observed mitochondrial gathering around the nucleus using fluorescence microscopy after gastric cancer cells were transfected with Mfn2-YFP (Figure 2a). For gastric cancer cells transfected with YFP-N1, the florescence diffused into the cytoplasm (Figure 2a). The efficiency of transfection was examined using flow cytometry. For gastric cell line SGC7901, Mfn2-YFP showed 41.3% ± 3.7% efficiency compared to 45.2% ± 2.6% for YFP-N1. For gastric cell line AGS, Mfn2-YFP showed 42.2% ± 4.1% efficiency compared to 44.3% ± 2.3% for YFP-N1. Western blot analysis (Figure 2b) confirmed that the expression of Mfn2 increased significantly after gastric cancer cells were transfected with Mfn2-YFP.

#### 2.1.3. Mfn2 Significantly Suppressed Gastric Cancer Cell Proliferation

**Figure 3:**
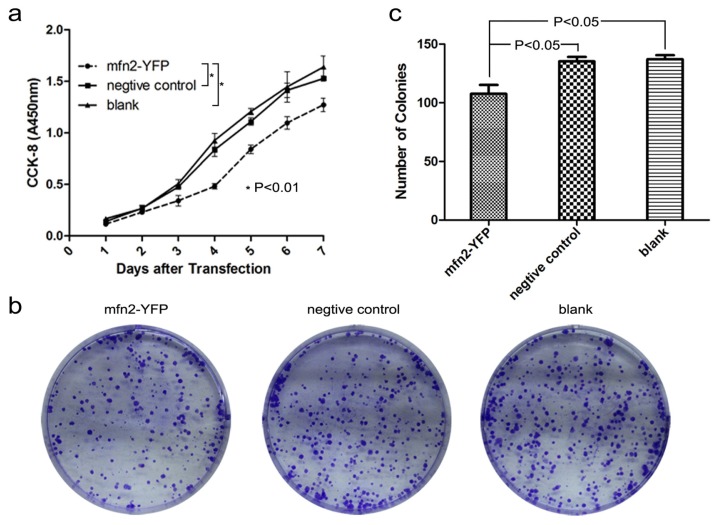
Mfn2 significantly suppressed gastric cancer cell proliferation. (**a**) The result of CCK-8 cell proliferation assay (detection: 450 nm, reference: 630 nm) showed the absorbance to be significantly lower in the Mfn2-YFP group compared to the other two groups (*p* < 0.01); (**b**,**c**) Colony-forming assay: The colony formation was significantly lower in the Mfn2-YFP group compared to the other two groups (*p* < 0.05). There was no difference between the negative control and blank (*p* > 0.05).

In cell proliferation assay (Figure 3a), the absorbance at 450 nm indicated that proliferation was significantly lower in the Mfn2-YFP group compared to the other two groups (*p* < 0.01). The proliferation curve indicated that cellular proliferation was suppressed by Mfn2. To confirm this, we performed a colony-forming assay (Figure 3b,c). After staining and counting, we found that colony formation was significantly lower in the Mfn2-YFP group compared to the other two groups (*p* < 0.05). There was no difference between the negative control and blank (*p* > 0.05).

#### 2.1.4. Mfn2 Halted the Cell Cycle in the G0/G1 Phase

**Figure 4:**
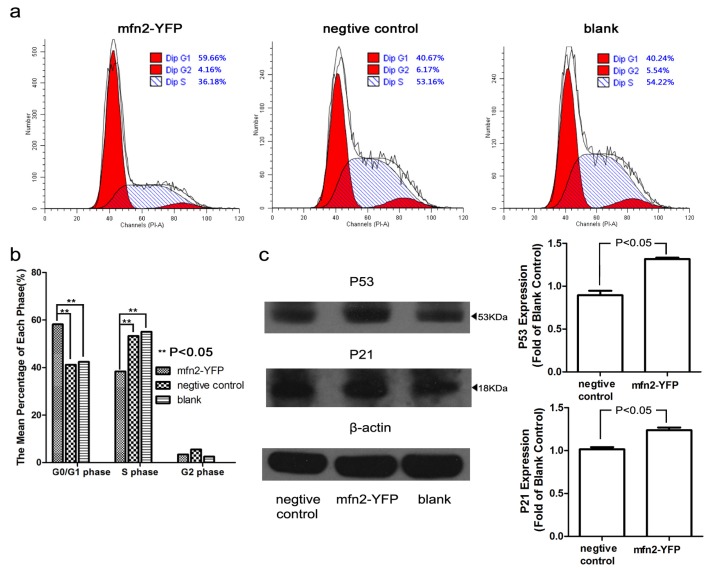
Mfn2 halted the cell cycle. (**a**,**b**) Typical images of cell cycle distribution. Mfn2 significantly increased the number of cells in the G0/G1 phase and reduced the number of cells in the S phase (*p* < 0.05, compared with both the negative control and blank); (**c**) Typical Western blot of P53 and P21 and their integrated density. The expression of P53 and P21 in the Mfn2-YFP group increased significantly.

To further explore how Mfn2 weakens proliferation, we stained cells with PI and analyzed the cell cycle by flow cytometry (Figure 4a,b). Mfn2 significantly increased the number of cells in the G0/G1 phase and reduced the number of cells in the S phase (*p* < 0.05, compared with both the negative control and blank). There was no significant difference between the negative control and blank. To explore the mechanism underlying this change, we analyzed proteins associated with the cell cycle, including cyclinA, cyclinB, cyclinD, cyclinE, CDK2, CDK4, CDK6, P53 and P21. We didn’t acquire significant change of cyclin A, B, D, E and CDK2, 4, 6 (data not shown). However, we found that P53 and its downstream protein, P21 [10], were increased significantly (Figure 4c). P21 may bind to and inhibit the various cyclin-CDK complexes [11,12]. This may be the reason why the cell cycle was affected.

#### 2.1.5. Mfn2 Induced Cell Apoptosis

**Figure 5:**
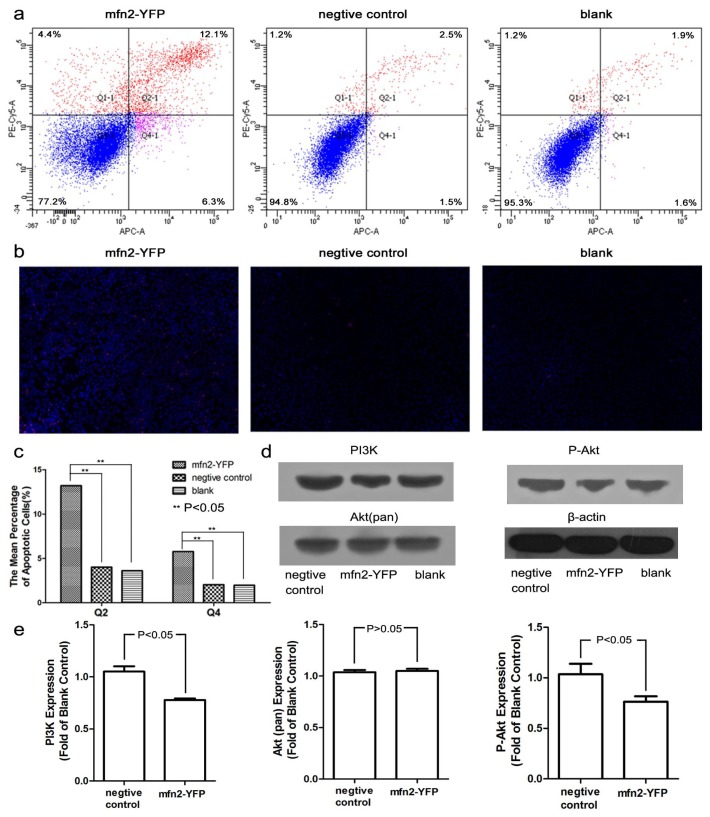
Mfn2 induced cell apoptosis. (**a**,**c**) Typical apoptotic images of flow cytometry. The numbers of early apoptotic cells (Q4 region) and late apoptotic cells (Q2 region) in Mfn2-YFP group increased significantly (*p* < 0.05, compared with both the negative control and blank); (**b**) Hoechst 33342/PI staining (apoptotic cells: blue ++, red +; necrotic cells: blue +, red ++; viable cells: blue +, red +; 200×). There were more bright blue cells (apoptotic cells) and bright red cells (necrotic cells) in the Mfn2-YFP group; (**d**,**e**) Typical Western blot of PI3K, Akt (pan) and phospho-Akt (P-Akt) and their integrated density. The expression of PI3K and phospho-Akt decreased in the Mfn2-YFP group.

Previous studies have reported that Mfn2 promotes apoptosis [7,8]. By flow detection (Annexin V-APC/PI double-labeled), we also confirmed that the numbers of late (Q2 region) and early apoptotic cells (Q4 region) were increased significantly (*p* < 0.05, compared with both the negative control and blank; Figure 5a,c). There was no difference between the negative group and blank (*p* > 0.05). Moreover, we observed a visible difference in Hoechst 33342/PI staining (Figure 5b). The cells in the Mfn2-YFP group were a brighter blue, and there were more necrotic (bright red) cells. This indicates that membrane permeability was enhanced (more Hoechst entered the cells), as occurs in apoptosis. Western blot analysis showed that PI3K and its main downstream protein, phospho-Akt, were decreased (Figure 5d,e). No significant change was found in Akt (pan). It has been shown that phospho-Akt can protect cells from apoptosis [13]. Thus, Mfn2-induced apoptosis was closely related with the decrease in phospho-Akt.

#### 2.1.6. The Invasion and Migratory Ability of Gastric Cancer Cells Was Weakened by Mfn2 Transfection

**Figure 6:**
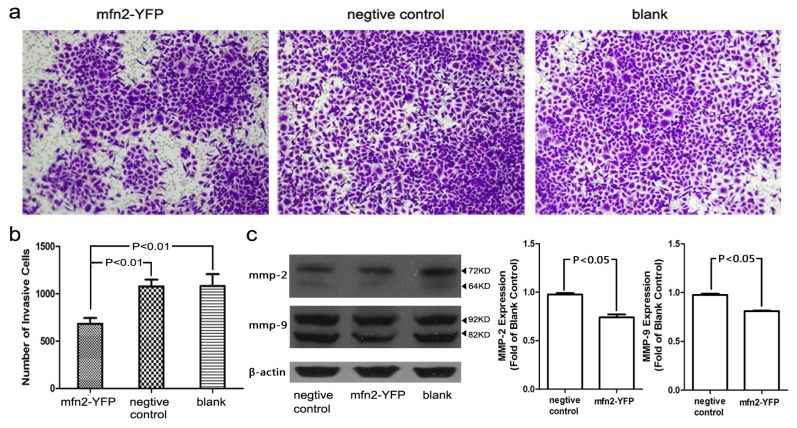
The invasion and migratory ability of gastric cancer cell line, AGS, was weakened by overexpression of Mfn2. (**a**,**b**) The number of cells that penetrated the membrane was less in the Mfn2-YFP group compared with the other two groups (*p* < 0.01); (**c**) Typical Western blot of MMP-2 and MMP-9 and their integrated density. The expression of MMP-2 and MMP-9 decreased after AGS was transfected with Mfn2-YFP.

A Transwell assay (Figure 6a,b) was conducted to study the influence of Mfn2 on cancer cell invasion and migration. BD Matrigel™ was used to imitate the extracellular matrix. The number of cells that penetrated the membrane was strongly correlated with the invasion ability of the cell line. Mfn2 weakened the invasion ability of AGS gastric cancer cells significantly (*p* < 0.01) compared with the negative control or blank, whereas there was no significant difference between the negative control and blank. We also observed the downregulation of MMP-2 and MMP-9 by Western blotting (Figure 6c). Low-level expression of MMP-2 and MMP-9, which promote both cell invasion and metastasis [14–17], may explain the reduced invasion and migratory ability we observed.

#### 2.1.7. Relationship between Mfn2 Expression and Clinicopathological Factors

We analyzed the relationship between Mfn2 expression in tumor tissue and various clinicopathological factors. We found a negative correlation between Mfn2 expression and tumor size (*p* < 0.05), indicating that the lower the expression of Mfn2, the more the tumor growth was out of control. No significant difference was found between Mfn2 expression and gender, age, adjacent organ invasion, lymphovascular invasion, differentiation degree, T stage, lymph node metastasis, distant metastasis or TNM (tumor, node, metastasis) stage (Table 1).

#### 2.1.8. Survival Analysis

**Figure 7:**
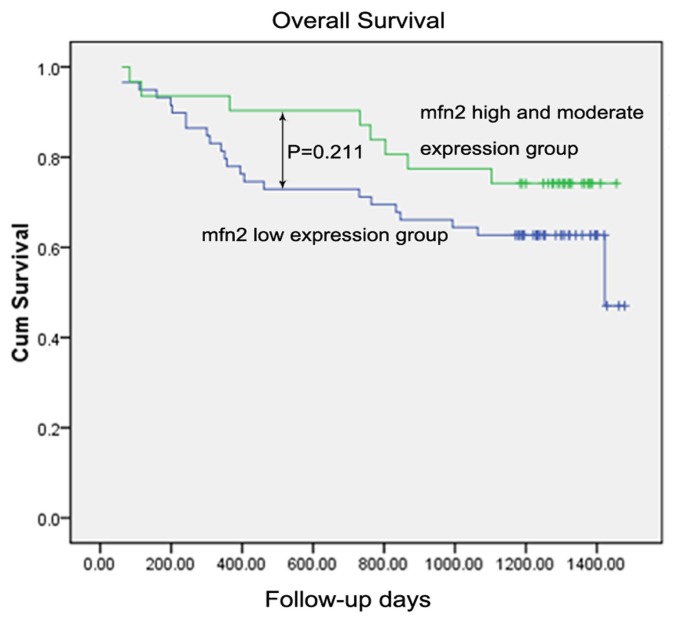
Kaplan-Meier survival curves according to the expression level of Mfn2 in 90 gastric cancer patients. The *p*-value between two groups was 0.211.

For the 90 patients, the one-year, two-year and three-year overall survival were 82.2%, 77.8% and 67.8%, respectively. In univariate analysis, the variables of age, tumor size, adjacent organ invasion, lymphovascular invasion, differentiation degree, T stage, distant metastasis and TNM stage acquired significant prognostic values on overall survival (OS) (all *p* < 0.05), while the Mfn2 expression showed no significance on overall survival (low expression *vs.* high and moderate expression, one-year OS 78% *vs.* 90.3%; two-year OS 71.2% *vs.* 87.1%; three-year OS 62.7% *vs.* 74.2%; *p* = 0.221) (Table 2, Figure 7). In order to evaluate the prognostic value of Mfn2 expression, we were forced to enroll it into multivariate analysis, together with factors of *p*-values less than 0.10 in univariate analysis. Finally, we identified adjacent organs invasion (*p* = 0.040; HR = 2.554; 95% CI 1.045–6.239), lymphovascular invasion (*p* = 0.015; HR = 2.563; 95% CI 1.204–5.459), distant metastasis (*p* = 0.002; HR = 8.817; 95% CI 2.242–34.679) and TNM stage III + IV (*p* = 0.009; HR = 3.730; 95% CI 1.395–9.970) as independent factors associated with worst overall survival (Table 2), while the remaining factors showed no prognostic significance (all *p*-values > 0.05, data not shown). However, Mfn2 expression still showed no significance on overall survival in multivariate analysis.

### 2.2. Discussion

Given recent advancements in medicine, especially the improved early diagnosis rate, the morbidity and mortality of gastric cancer have declined in many advanced countries. However, these advances have not changed the treatment of gastric cancer by much. Surgery remains the most efficient therapy, but a high survival rate cannot be attained with advanced stage patients, and chemical therapy and radiotherapy lack specificity. Recently, with the development of molecule-targeted therapy, scientists have focused increasingly on gastric cancer treatment. Many relevant target molecules or pathways have been identified, including epidermal growth factor receptor, vascular endothelial growth factor receptor, fibroblast growth factor receptor, insulin-like growth factor receptor, the PI3k/Akt/mTor pathway and the c-Met pathway [18]; however, these target molecules are less than satisfactory. Therefore, it is critical that we find more specific and more useful genes or biomarkers. In our research, we investigated Mfn2, which has been studied widely in CMT disease. Researchers have reported that Mfn2 is correlated with anti-tumor activity in several malignancies [7,8]. The current study confirmed such an association in gastric cancer cell lines, and we found a negative relationship with tumor size.

Mfn2, which controls mitochondrial fusion, is a highly conserved GTPase [19]. Similar proteins are found in yeasts, *Caenorhabditis elegans*, fruit flies and mammals [20]. Mfn2 possesses two transmembrane domains spanning the outer mitochondrial membrane, a possible PKA/PKG phosphorylation site and a p21 (Ras) signature motif (amino acids 77–92), which plays an important role in signaling [6,19].

Previous studies have demonstrated that the mutation of Mfn2 can cause CMT neuropathy type 2A [21,22]. Additionally, reduced expression of Mfn2 plays an important role in insulin resistance [23]. One recent study revealed that Mfn2 markedly reduced the proliferation of VSMCs [6]; further research confirmed this anti-proliferative effect in many tumor cell lines, including hepatocellular carcinoma cell line, HepG2, urinary bladder carcinoma cell lines, T24 and 5637, lung cancer cell line, A549, and colon cancer cell line, HT-29 [8,9,24]. Moreover, in BM-1 cells, the anti-proliferative effect of Mfn2 has been shown to be stronger than that of the anti-tumor gene, p53 [6].

In the current study, we demonstrated that Mfn2 suppressed the proliferation of SGC7901 gastric cancer cells using both a CCK-8 cell proliferation assay and colony-forming assay. Moreover, we confirmed that Mfn2 expression was higher in normal gastric mucosa than in tumor tissue, as shown by QRT-PCR, Western blotting and immunohistochemical analysis. The expression of Mfn2 in tumor tissue was negatively related to tumor size. Taken together, we have reason to consider Mfn2 to be a potential anti-tumor gene. Meanwhile, we did not observe a significant difference between the expression of Mfn2 and TNM stage or clinical prognosis, though the one-year, two-year and three-year overall survival of the Mfn2 low-expression group was lower than the moderate and high-expression group. However, the findings may have been affected by limited sample size or follow-up time.

We found that Mfn2 caused a cell cycle arrest in the G0/G1 phase. The cell cycle consists of four distinct phases (G1, S, G2 and M). Each phase is regulated by its relevant cyclins, CDKs and CDK inhibitors (CDKIs). The p21 (Ras) signature motif in the N-terminal region of Mfn2 is important for its regulation of the cell cycle. A yeast two-hybrid assay confirmed that Mfn2 interacts with a Ras analog [6]. This was validated by the co-immunoprecipitation of Mfn2 with Ras [6]. Our Western blot analysis revealed a p53 and p21 rise in SGC7901 cells transfected with Mfn2. Scientists have studied p53 for many years, and its close relationship with apoptosis has been described [25]. P21, as a downstream partner of p53, could be regulated by p53. P21 is a CDKI that binds cyclin-CDK complexes, and it has been shown to be necessary for a p53-mediated G1 arrest in human cancer cells [11,26]. In our experiment, we also observed a significant increase of the G0/G1 phase. The cells in the G1 phase will synthetize cyclin D, which interacts with CDK4 and CDK6. These active complexes initiate phosphorylation of the members of the retinoblastoma (Rb) family. This leads to transcription of the gene of cyclin A and E. Cdk2/cyclin E complexes are active at the G1/S transition and promote entry into the S phase [27]. Hence, we conjectured that the increased p21 would bind to and inhibit the activity of cyclin D/CDK4, cyclinD/CDK6 or cyclin E/CDK2 complexes, leading to cell cycle arrest.

In addition to blocking the cell cycle, we demonstrated that Mfn2 induced apoptosis. Karbowski [28] implicated Mfn2 in the regulation of mitochondrial morphology and showed that it colocalized with the proapoptotic molecule, Bax, on mitochondria, indicating a potential regulatory function in apoptosis. Our studies confirm that Mfn2-induced apoptosis is likely to occur through the PI3K-Akt pathway, because our Western blot results showed that both PI3K and phospho-Akt were reduced. Previous reports have indicated that a signaling pathway from PI3K to the serine/threonine protein kinase, Akt, might mediate some cellular responses of PI3K, including protection from apoptosis [13]. Akt is a direct effector of PI3K that is activated by phosphorylation. The mechanism by which Akt suppresses apoptosis, however, is unknown. For example, activated Akt can phosphorylate BAD. BAD is a distant member of the Bcl-2 family that promotes cell death; however, the phosphorylation of BAD prevents this [29,30]. Also, the overexpression of active Akt could successfully block ceramide-induced neuronal cell death by inhibiting the translocation of the apoptosis-inducing factor [31]. Moreover, in a rat model of cerebral ischemia, Akt was suggested to phosphorylate JNK, thereby suppressing the pro-death JNK pathway and promoting neuron survival [32]. Lastly, Akt could inhibit GSK3 by phosphorylating it at Ser21 and Ser9, causing an anti-apoptotic effect [33]. Considering these potential mechanisms together, we suggest that reduced PI3K and phospho-Akt levels play an important role in Mfn2-induced apoptosis.

Akt is also associated with cell invasion and migratory abilities. Kim [34] demonstrated that Akt potently promoted HT1080 cell invasion by increasing cell motility and MMP-9 production; the increase in MMP-9 production was mediated by the Akt-induced activation of nuclear factor-kappaB transcriptional activity. Adya [35] also demonstrated that the inhibition of PI3K/Akt signaling led to significant decreases in visfatin-induced MMP and VEGF production and activation, along with significant reductions in endothelial proliferation and capillary tube formation. The key role of MMPs in tumor invasion and metastasis has been well established [15]. Interest in these enzymes is based on the capacity of these enzymes to degrade type IV collagen, a major component of basement membranes [17]. Masson [36] demonstrated for the first time in an experimental model that MMP-2 and MMP-9 cooperated to promote the *in vivo* invasive and angiogenic phenotype of malignant keratinocytes. In our study, the expression of MMP-2 and, especially, MMP-9 was significantly reduced. As a result, the invasion ability of AGS cells was suppressed by Mfn2 in a transwell assay. We further discovered that PI3K and phospho-Akt were reduced following transfection with Mfn2. These findings indicate that the downregulation of phospho-Akt may have diminished the expression of MMP-2 and MMP-9, thereby reducing the invasion ability of gastric cancer cells.

## 3. Experimental Section

### 3.1. Patient Samples

Between February 2009 and December 2009, a total of 90 patients diagnosed with gastric cancer, none of whom received radiotherapy or chemotherapy before surgery, at the Department of Gastrointestinal Surgery, First Affiliated Hospital, Medical College, Zhejiang University (Zhejiang, China), were enrolled in this study. Paired tumor tissue and normal mucosal tissue samples were obtained from each patient during surgery. The specimens were stored at −80 °C until use and were embedded in paraffin. Prior to their participation in the study, informed consent was obtained from all patients and appropriate permission was granted by the ethical committee of the First Affiliated Hospital, Medical College, Zhejiang University.

### 3.2. Materials

Plasmid Mfn2-YFP, containing the complete Mfn2 ORF (2282 bp) was a gift from Richard Youle (Addgene plasmid 28010). Empty YFP-N1 vector was used as a negative control. The human gastric cancer cell lines, SGC7901 and AGS, were obtained from the Chinese Academy of Medical Sciences and Shanghai Institute for Biological Sciences, respectively. Cells were cultured in RPMI-1640 medium (Thermo Fisher Scientific, Beijing, China) with 10% fetal bovine serum (FBS) (SAFC Brooklyn, Melbourne, Australia) and maintained in an incubator at 37 °C with 5% CO_2_.

### 3.3. Immunohistochemistry

Immunohistochemical analysis was carried out using paired paraffin-embedded cancer tissues and normal mucosal samples. Briefly, serial 4 μm sections were deparaffinized and rehydrated, then subjected to heat-induced epitope retrieval in a microwave oven. Next, the sections were treated with 3% hydrogen peroxide to quench endogenous peroxidase activity, followed by incubation with 5% FBS to block nonspecific binding. After blocking, the sections were incubated overnight at 4 °C with primary monoclonal antibodies (mouse anti-Mfn2, 1:400 (Abcam, Cambridge, UK)). The next day, the sections were incubated with HRP-conjugated secondary antibodies (Invitrogen, Carlsbad, CA, USA) and visualized using 3,3′-diaminobenzidine (Zhongshan Golden Bridge Biotechnology, Beijing, China), followed by counterstaining with hematoxylin. For the negative control, the primary antibody was replaced with phosphate-buffered saline (PBS).

The stained slides were scored independently by two observers in a blinded manner under microscope (BX41; Olympus, Tokyo, Japan). As the expression of Mfn2 was universal, the scores were determined by grading the staining intensity as follows: low expression (+, weak staining, slightly stronger than background staining); moderate expression (++, moderate staining, yellow brown); high expression (+++, strong staining, brown).

### 3.4. Western Blotting

Total protein was extracted from tissues or cells (48 h after transfection) using RAPI lysis buffer (Beyotime, Jiangsu, China) containing 1 mM PMSF. SDS-PAGE was used to separate the extracted proteins, followed by transfer to PVDF membranes. After blocking with 5% non-fat milk, the membranes were incubated overnight on ice with primary antibodies against Mfn2 (1:1000), matrix metalloproteinase (MMP)-2 (1:1000), MMP-9 (1:1000), P53 (1:1000), P21 (1:200), cyclin-dependent kinase (CDK)2, CDK4, CDK6 (all dilutions 1:500), PI3K (1:1000), cyclinB, cyclinD, cyclinE (all dilutions 1:1000) (Abcam), cyclinA (1:200; Santa Cruz Biotechnology, Santa Cruz, CA, USA), Akt (pan, 1:1000; Cell Signaling Technology, Beverly, MA, USA) and phospho-Akt (Ser 473, 1:1000; Cell Signaling Technology, Beverly, MA, USA). The next day, the membranes were washed with TBST and incubated with HRP-conjugated anti-rabbit and anti-mouse secondary antibodies for 2 h at room temperature. Next, a membrane-enhanced chemiluminescence reagent ECL kit (Israel Beit Haemek Ltd., Beit Haemek, Israel) was used to detect the immunoreactive bands on the blots. β-Actin (1:1000; Sigma-Aldrich, St. Louis, MO, USA) was used as an internal control.

### 3.5. Quantitative Real-Time PCR (QRT-PCR)

We performed QRT-PCR (7500Fast; ABI, Foster City, CA, USA) to compare the mRNA expression of Mfn2 in 30 paired tumor tissue and normal mucosal tissue samples from gastric cancer patients (selected at random). The primers used were 5′-CCCCCTTGTCTTTATGCTGATGTT-3′ and 5′-TTTTGGGAGAGGTGTTGCTTATTTC-3′.

### 3.6. Transfection

The plasmid was transfected into cells using X-tremeGENE HP DNA Transfection Reagent (Roche, Mannheim, Germany), according to the manufacturer’s instructions. The efficiency of transfection was tested by flow cytometry and Western blot analysis.

### 3.7. Cell Proliferation and Colony Formation

SGC7901 cells were seeded into 96-well plates at a density of 2000 cells per well, grown at 37 °C overnight and then transfected with Mfn2-YFP (experimental group) or YFP-N1 (negative control) or treated with transfection reagent alone (blank). Cell proliferation was tested using a CCK-8 Kit (Dojindo Laboratories, Kumamoto, Japan) every 24 h after transfection for 7 days (the reactions were incubated for 1 h at 37 °C and 5% CO_2_; detection: 450 nm, reference: 630 nm). A colony-forming assay was included to further test cell viability. After transfection, the cells were digested and counted using an automated cell counter (Vi-CELL; Beckman Coulter, Inc., Brea, CA, USA). Briefly, 1000 viable cells were seeded in 6-well plates and cultured at 37 °C with 5% CO_2_ for 10–14 days. Next, the colonies were stained with crystal violet and counted. All experiments were repeated three times.

### 3.8. Cell Cycle and Apoptosis

The cell cycle was analyzed using a Cell Cycle Detection Kit (KeyGEN, Nanjing, China). Before transfection, we synchronized the cell cycle by serum-free medium. At 48 h after transfection, the cells were digested and washed with PBS, then fixed in 70% alcohol overnight. The next day, the cells were washed and incubated with RNase A at 37 °C for 30 min and then incubated with propidium iodide (PI) at 4 °C for another 30 min. The samples were examined by flow cytometry (Becton-Dickinson, Franklin Lakes, NJ, USA).

Cell apoptosis was analyzed using a Hoechst 33342/PI Detection Kit (KeyGEN, Nanjing, China). We stained cells after transfection in 6-well plates with Hoechst 33342 and PI successively and then acquired images under a fluorescence microscope (IX81, Olympus, Tokyo, Japan). Cells were also digested and tested by flow cytometry using an Annexin V-APC/PI Apoptosis Analysis Kit (Tianjin Sungene Biotech Co., Tianjin, China). All experiments were repeated three times.

### 3.9. Cell Migration and Invasion

A transwell (Millipore, Basel, Switzerland) assay was used to estimate the influence of Mfn2 on migration and invasion by gastric cancer cells. Because the invasion ability of SGC7901 cells was weak, we selected AGS cells for our experiment and we used BD Matrigel™ Matrix (BD Bioscience, Bedford, MA, USA) to coat 8.0 μm transwells. After transfection, 3 × 10^5^ cells were seeded with serum-free RPMI-1640 into each transwell, with 10% FBS RPMI-1640 in a 24-well plate. After 48 h, the transwell membrane was washed, fixed and stained. Finally, images were acquired using a BX41 microscope (Olympus, Tokyo, Japan). All experiments were repeated three times.

### 3.10. Statistical Analysis

The results are expressed as the mean ± SD. A two-tailed, paired Student’s *t*-test was used to analyze numerical variables. The Wilcoxon signed-rank test or Chi-square test was used to analyze our immunohistochemical results and correlations with the clinicopathological factors. The follow-up period commenced at the start of surgery. Overall survival was calculated from the date of surgery until death or last contact. Potential prognostic factors, including gender, age, tumor size, adjacent organs invasion, lymphovascular invasion, differentiation degree, TNM stage and Mfn2 expression were entered into a univariate analysis using the Kaplan-Meier product-limit method and compared using the log-rank test. Prognostic factors with *p*-values of <0.10 in the univariate analysis were enrolled into Cox proportional hazard regression models with the forward likelihood method to determine which variables were associated independently with overall survival. Statistical analyses were performed using SPSS software (ver. 16.0 for Windows; SPSS, Chicago, IL, USA). A value of *p* < 0.05 was considered to indicate statistical significance. ImageJ software was used to quantify the results of Western blotting. GraphPad Prism 5.0 software (Version 5.01, GraphPad Software, Inc. La Jolla, CA, USA, 2009) was used to create artwork.

## 4. Conclusions

In summary, we demonstrated that the expression of Mfn2 was lower in tumor tissue than in normal mucosal tissue in gastric cancer patients and that it was negatively correlated with tumor size. We also confirmed that Mfn2 suppressed cell proliferation, arrested the cell cycle in the G0/G1 phase, induced apoptosis and weakened the invasion ability of gastric cancer cells. Therefore, Mfn2 is a potential anti-tumor gene and a potential therapeutic target for the treatment of gastric cancer. If we could control the expression of Mfn2, it is possible that the progression of gastric cancer could be delayed.

## Figures and Tables

**Table 1:** Correlation of Mfn2 expression (IHC) with clinicopathological factors in gastric cancer.

	Mfn2 expression in tumor tissue			
		
Clinicopathological factors	Number (*n* = 90 )	Low (*n* = 59)	High and moderate a (*n* = 31)	*p-*value
*Gender*				

Male	64	41	23	0.640
Female	26	18	8	

*Age*				

>65	34	22	12	0.895
≤65	56	37	19	

*Tumor size*				

<4 cm	47	26	21	0.033 b
≥4 cm	43	33	10	

*Adjacent organ invasion*				

Yes	10	7	3	1.000
No	80	52	28	

*Lymphovascular invasion*				

Yes	27	16	11	0.411
No	63	43	20	

*Degree of differentiation*				

Well and moderate	18	12	6	0.912
Poor	72	47	25	

*T stage*^c^				

T1 + T2	26	17	9	0.983
T3 + T4	64	42	22	

*Lymph node metastasis*^c^				

Yes	65	44	21	0.492
No	25	15	10	

*Distant metastasis*^c^				

M0	87	56	31	0.510
M1	3	3	0	

*TNM stage*^c^				

I + II	36	22	14	0.469
II + IV	54	37	17	

a Because there were only three samples of tumor tissue with strong staining (+++), we classified it into the same group with moderate staining (++);

b Significant at the *p* < 0.05 level;

c Sixth Edition of the American Joint Committee on Cancer. All data were analyzed by the Chi-square test.

**Table 2:** Univariate and multivariate analyses regarding overall survival in 90 gastric cancer patients.

Factors	Number (*n* = 90)	Univariate analysis a*p*-value	Multivariate analysis b	

			HR (95%CI)	*p*-value
*Gender*				

Male	64	0.492		
Female	26			

*Age*				

>65	34	0.021		
≤65	56			

*Tumor Size*				

<4cm	47	0.008		
≥4cm	43			

*Adjacent organ invasion*				

Yes	10	<0.001	2.554 (1.045–6.239)	0.040
No	80		1	

*Lymphovascular invasion*				

Yes	27	0.036	2.563 (1.204–5.459)	0.015
No	63		1	

*Degree of differentiation*^c^				

Well and moderate	18	0.022		
Poor	72			

*T stage*^d^				

T1 + T2	26	0.001		
T3 + T4	64			

*Lymph node metastasis*^d^				

Yes	65	0.064		
No	25			

*Distant metastasis*^d^				

M0	87	<0.001	1	
M1	3		8.817 (2.242–34.679)	0.002

*TNM stage*^d^				

I + II	36	0.001	1	
III + IV	54		3.730 (1.395–9.970)	0.009

*Mfn2 expression in tumor tissue*				

Low expression	59	0.211		
High and moderate expression	31			

a Univariate analysis was performed by Kaplan-Meier analysis model and the log-rank test. Variables of *p* < 0.10 in the univariate analysis were enrolled into a multivariate analysis;

b Multivariate analysis was performed by the Cox proportional hazards model with the forward likelihood method;

c Degree of differentiation: ring cell carcinoma was included in the poor differentiation group;

d Sixth Edition of the American Joint Committee on Cancer.
